# Supplementary figures and images for: The CHD family chromatin remodeling enzyme, Kismet, promotes both clathrin-mediated and activity-dependent bulk endocytosis
Source: PLoS One. 2024 Mar 21;19(3):e0300255. doi: 10.1371/journal.pone.0300255 (PMC10956772; doi:10.1371/journal.pone.0300255)

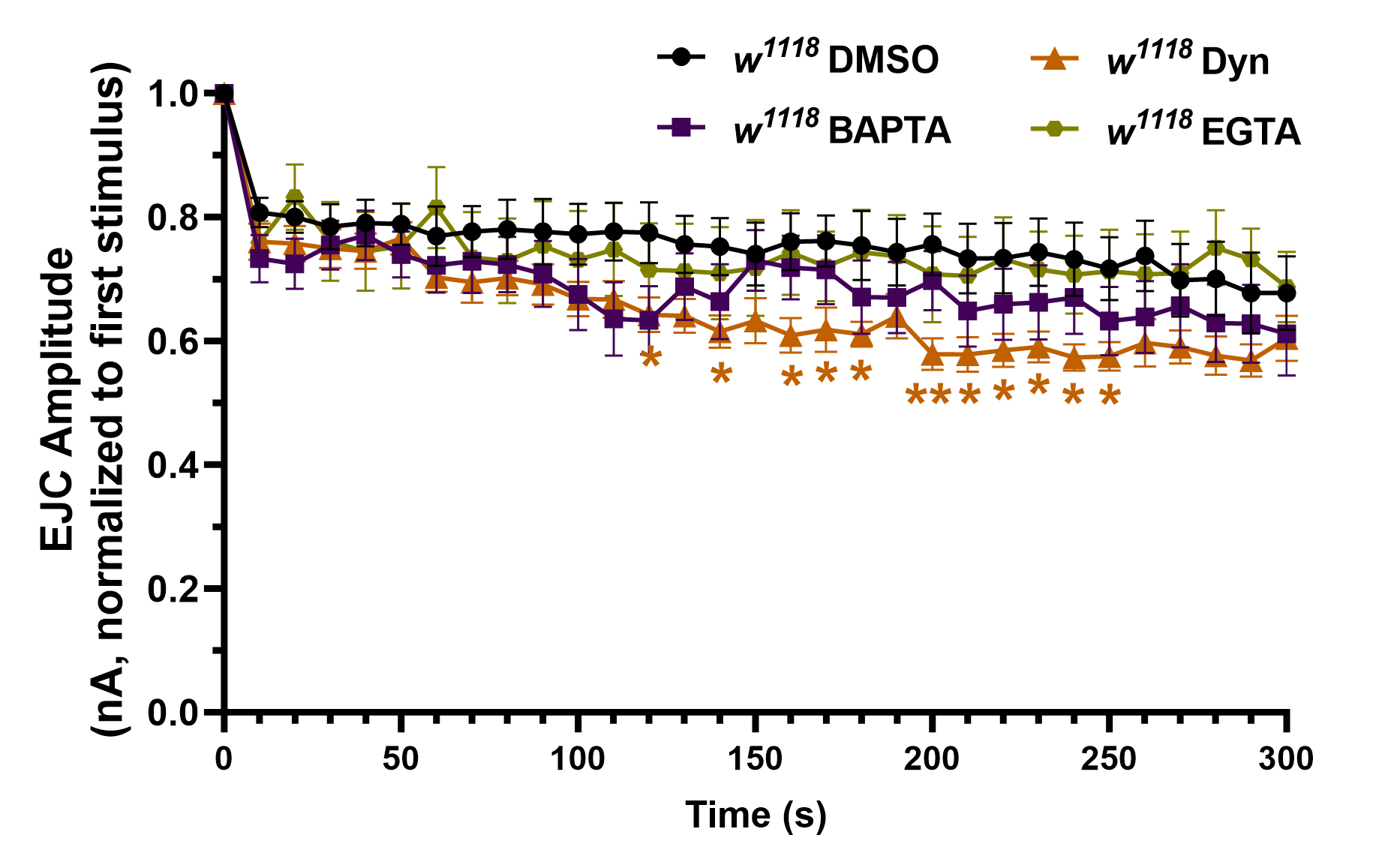

Supplement: S1 Fig — eEJCs were recorded during 5 Hz stimulation for five minutes in HL-3 + 1.0 mM Ca2+. 100 μM BAPTA (n = 10), 25 μM EGTA (n = 10), 100 μM Dynasore (n = 9), or an equal volume of DMSO (controls, n = 11) were applied for 10 minutes prior to neuronal stimulation. Each eEJC is normalized to the first stimulus for each condition. Points represent mean relative eEJC amplitudes. Error bars represent the SEM. (TIF) [file pone.0300255.s001.tif]

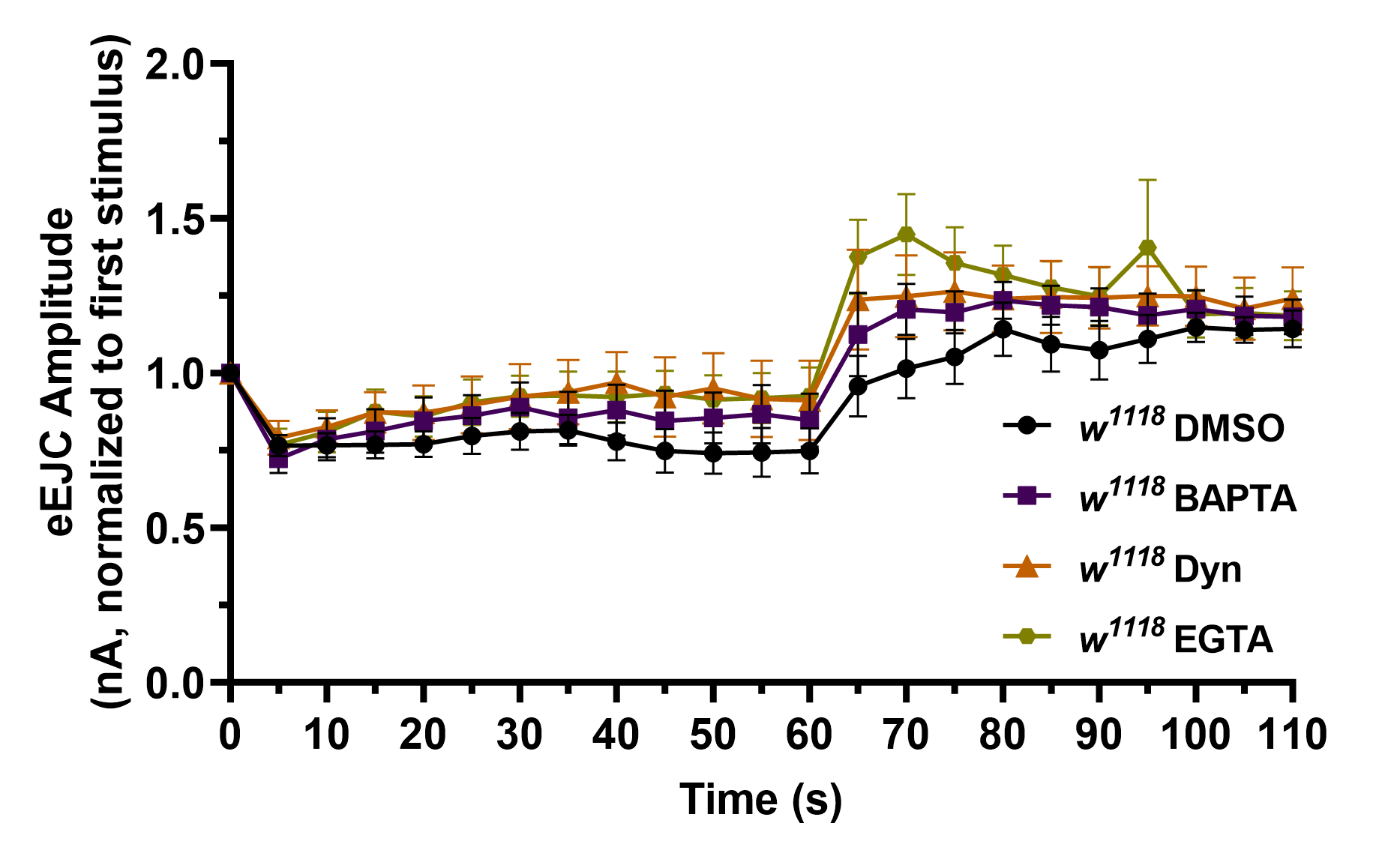

Supplement: S2 Fig — eEJCs were measured in HL-3 + 1.0 mM Ca2+ for 60 sec of 20 Hz HFS to induce ADBE and during a 50 sec recovery period with 0.2 Hz stimulation. 100 μM BAPTA (n = 10), 25 μM EGTA (n = 9), 100 μM Dynasore (n = 9), or an equal volume of DMSO (controls, n = 10) were applied for 10 minutes prior to neuronal stimulation. Each eEJC is normalized to the first stimulus for each condition. Points represent mean relative eEJC amplitudes. Error bars represent the SEM. (TIF) [file pone.0300255.s002.tif]

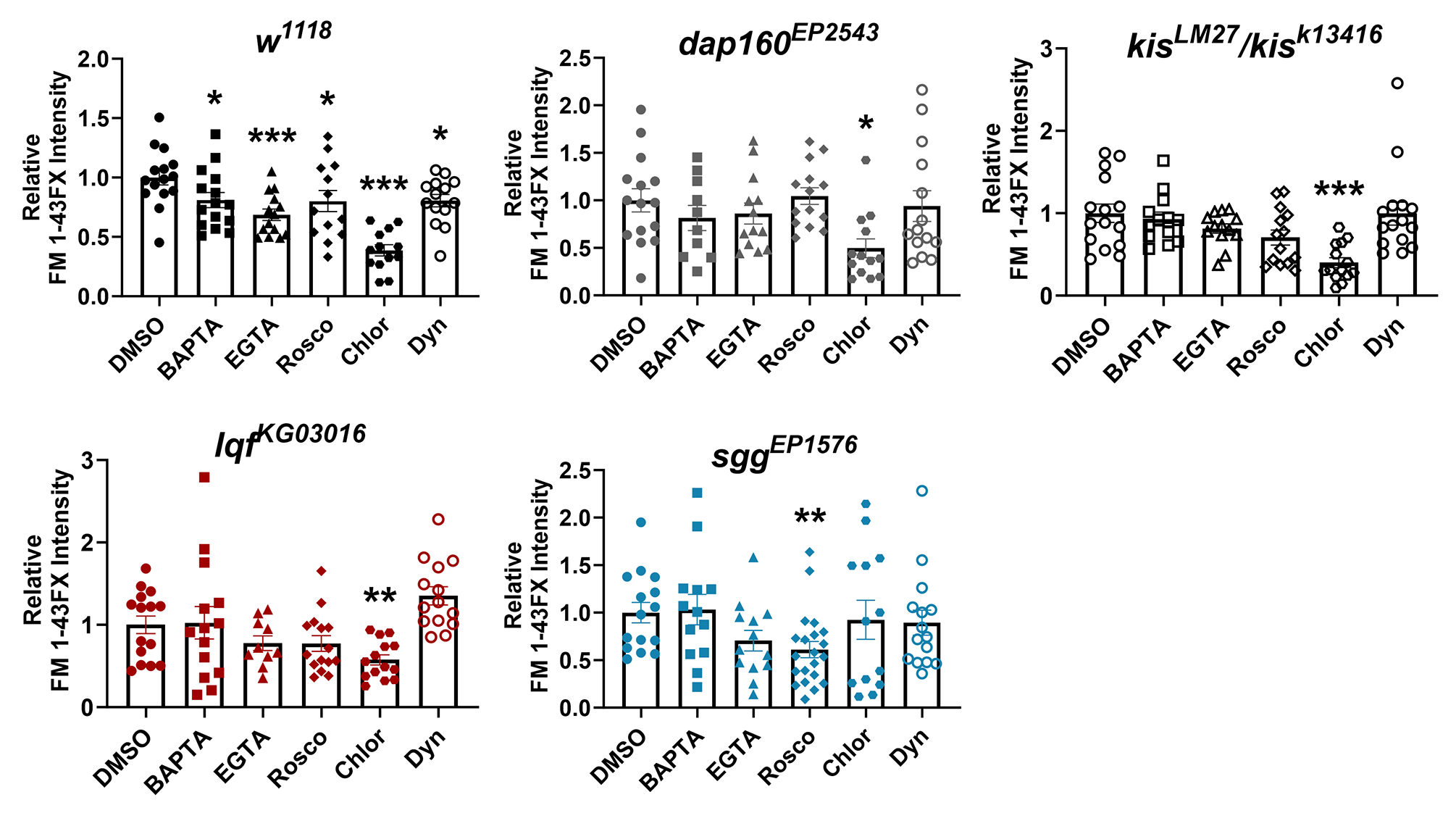

Supplement: S3 Fig — Endocytosis was assessed by measuring internalization of the lipophilic dye FM 1-43FX after one min stimulation with 90 mM KCl. Panels show high resolution confocal micrographs of terminal presynaptic motor neuron boutons (HRP, magenta) after internalization of the lipophilic dye FM 1-43FX (green). Data for each genotype were normalized to the DMSO control condition. (TIF) [file pone.0300255.s003.tif]

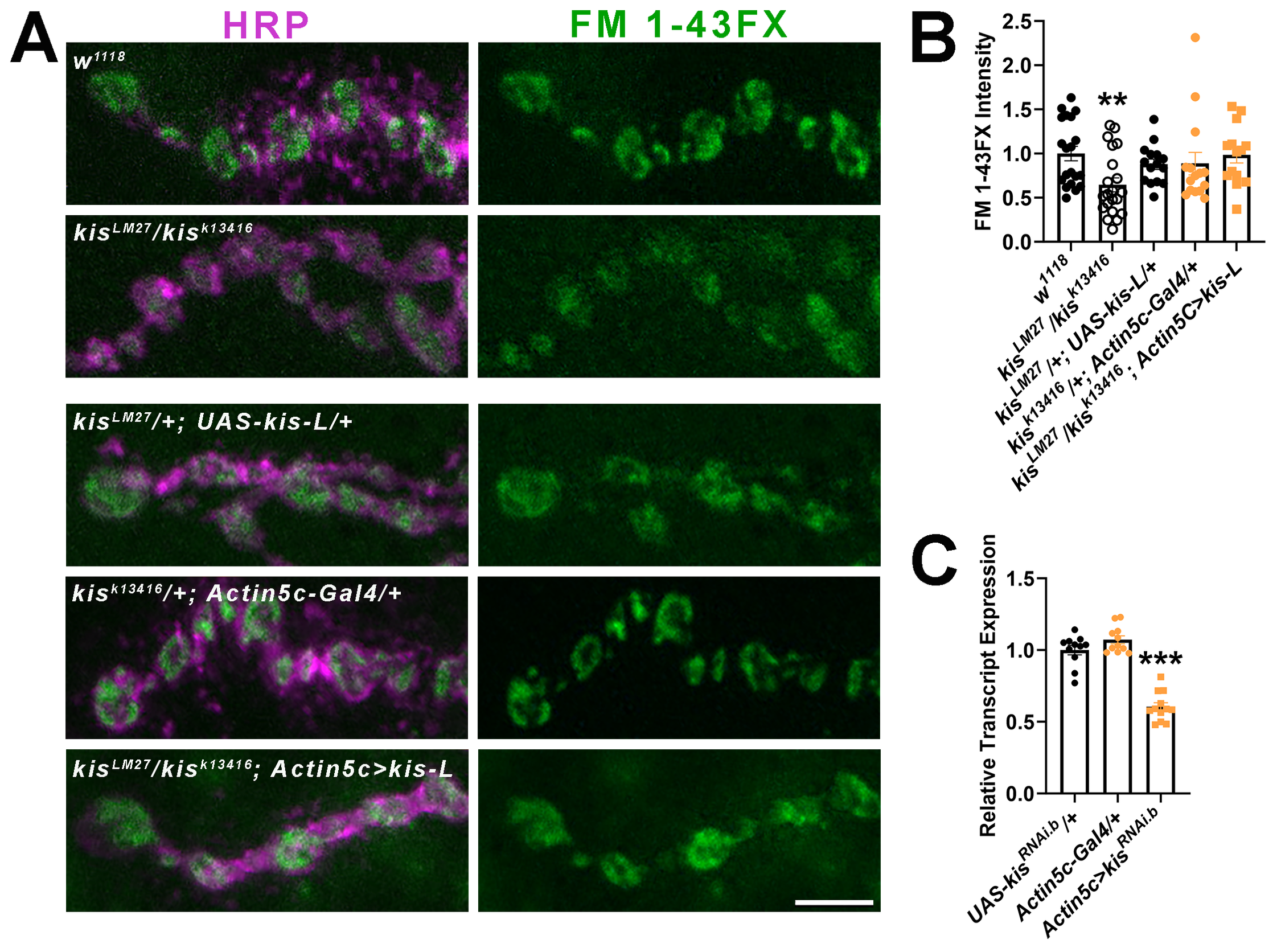

Supplement: S4 Fig — The Actin5c-Gal4 driver was used to express UAS-kis-L in all tissues of kis mutants. A) High resolution confocal micrographs of terminal presynaptic motor neuron boutons (HRP, magenta) after internalization of the lipophilic dye FM 1-43FX (green) after one min stimulation with 90 mM KCl in genotypes as listed. Scale bar = 5 μm. B) Quantification of FM 1-43FX fluorescence. C) Relative expression of CNS transcripts was assessed via RT-qPCR. 2-ΔΔC(t) values are indicated. Data includes four biological replicates each including three technical replicates. Technical replicates are represented by the points for the representative genotypes. Bars indicate the SEM. (TIF) [file pone.0300255.s004.tif]

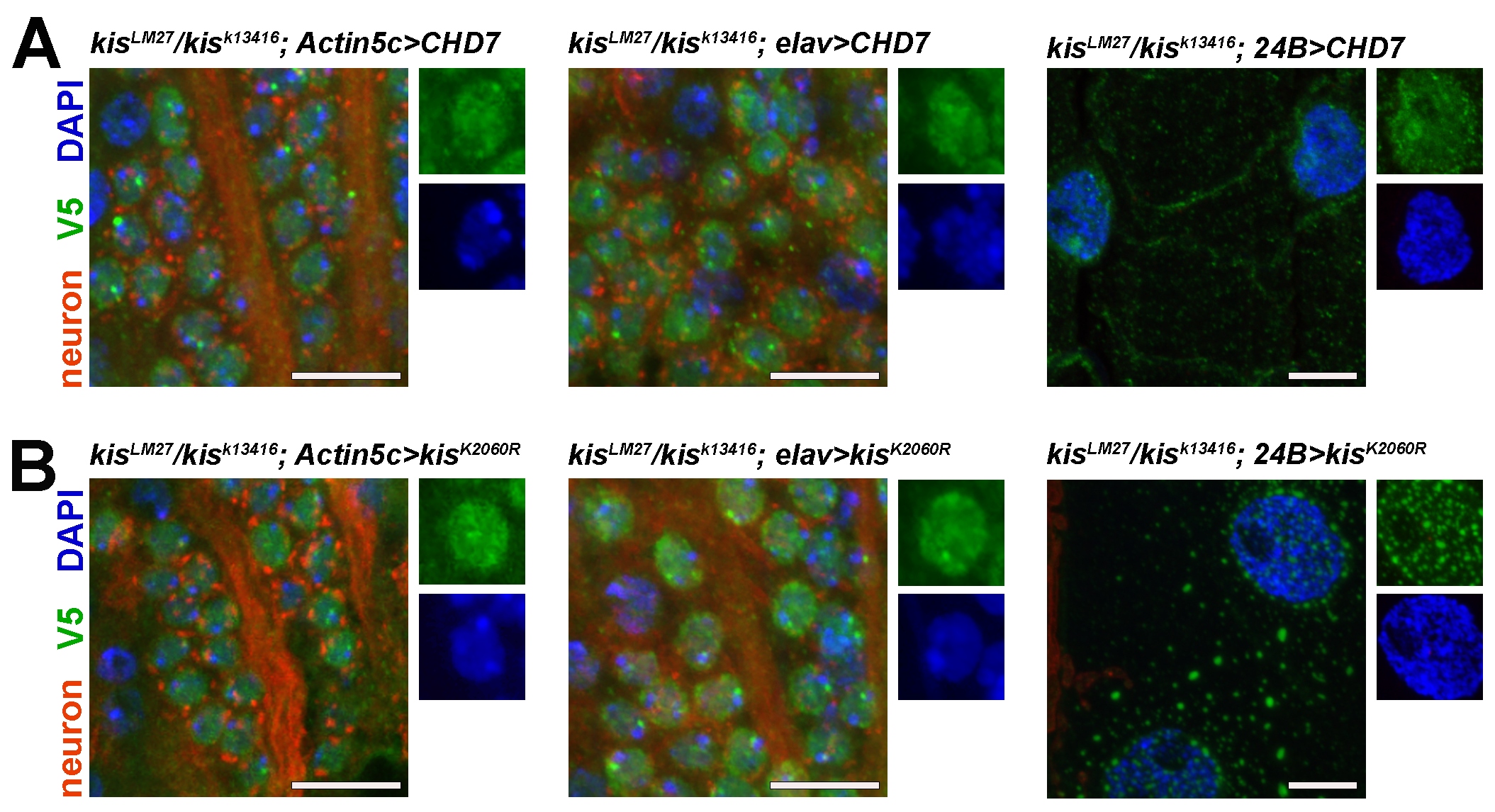

Supplement: S5 Fig — Confocal micrographs showing V5 (green) and HRP (magenta, neuron) immunolabeling and DAPI labeling. Left and middle large panels show representative ventral nerve cords with single nuclei depicted in the small panels of kis mutants expressing human CHD7 (A) or the ATPase deficient KisK2060R (B). Scale bar = 5 μm. Right panels show representative muscles with single nuclei depicted in the small panels of kis mutants expressing human CHD7 (A) or the ATPase deficient KisK2060R (B). Scale bar = 5 μm. (TIF) [file pone.0300255.s005.tif]
