# Supplementary material for: The CHD family chromatin remodeling enzyme, Kismet, promotes both clathrin-mediated and activity-dependent bulk endocytosis
Source: PLoS One. 2024 Mar 21;19(3):e0300255. doi: 10.1371/journal.pone.0300255 (PMC10956772; doi:10.1371/journal.pone.0300255)
Supplement: Attachment — Submitted filename: Response to Reviewers.docx [file pone.0300255.s007.docx]

We thank the reviewers for the time they dedicated to carefully reviewing our manuscript. We appreciate their insightful comments and are confident that their feedback has improved the manuscript. Reviewer comments are shown in normal text. Our responses are in blue.

**Reviewer #1:** In this work, the authors investigate the role of the chromatin remodeling enzyme Kismet in synaptic endocytosis at Drosophila neuromuscular junctions, an excellent and popular system for studying molecular mechanisms of synaptic vesicle endocytosis. The work builds on previous publications by the authors showing that kismet is important for recycling synaptic vesicles during endocytosis.
In extensive experiments using *kis* mutants and rescue and RNAi approaches, they show that Kismet promotes clathrin-mediated and activity-dependent mass endocytosis of synaptic vesicles. They also show that kismet is important in postsynaptic muscle to promote presynaptic endocytosis as well as locomotion. Interestingly, this function seems to be independent of the ATPase domain.
Although the molecular mechanism of how kismet mediates these processes is still unclear, this work represents an important and novel contribution to the study of the physiological function of kismet. The experiments seem to be carefully executed, however, some points, especially related to evaluation of experiments and statistical analysis, need to be addressed in more detail.

Major points:

1) Experimental details, sample size: Throughout the manuscript, there is no indication of how many animals/samples were used for each experiment. It is only stated in general terms that at least two independent biological experiments were performed. In addition, it is not clear what the sample points shown present. For example, in the endocytosis experiments, does a sample point represent the analysis of a section or a projected z-stack slice? How many sections per larva and how many larvae were used? Electrophysiology - how many experiments were performed with how many larvae? Is the mean value given? What about error bars? All this is of course important for the later statistical analysis and must therefore be indicated in the legends to the figures of the individual experiments.

The sample points shown represent the sample size. This information was included in the methods:

Bar graphs in figures show sample sizes. Statistical significance is represented on bar graphs as follows: * = <0.05, ** = <0.01, *** = <0.001 with error bars representing standard error of the mean (SEM). Table S1 shows the statistically significant p - values corresponding to each figure.

However, we have amended the text in the methods to include that sample points for endocytosis experiments represent the mean of the z-stack slices and means are always shown in the graphs. We have also added this information to the figure legends for the figures that show electrophysiological data. For all experiments, the number of Z-sections vary from larva to larva as the depth of the NMJ varies from animal to animal. There was also a supplemental table included to show p-values for all experiments.

2) Statistics: The authors state that they used an unpaired t-test when there was only one control. This is not correct, because for experiments with more than two groups, the comparison must be made with a one-way ANOVA and an appropriate post-hoc test. This is independent of the number of control groups. Using an ANOVA you can be confident that any statistically significant result you find is not just due to running many tests. In addition, authors should check for normality distribution.

Our statistics were performed as the reviewer described. Unpaired t-tests were used, for example, to compare the *kis* mutant to its *w^1118^* control. For any experiment that included more than one control, and therefore more than two groups, one-way ANOVAs were performed with post hoc analyses. Post hoc tests utilized were determined by the “normality distribution”, which was indicated by Bartlett’s test for homogeneity of variances. Because the reviewer raised this concern, we amended the “Experimental Design and Statistical Analyses” section of the methods to clarify this.

3) Quantification of endocytosis: as I understand it, the authors measured FM 1-43X intensity. Can the authors rule out an effect of the genetic manipulations/treatments on the size/area of the boutons and thus on the fluorescence intensity of FM 1-43X? Have they also measured HRP fluorescence intensity and is it comparable between NMJs?

Although there are not published differences in bouton sizes for any of the genotypes used in our experiments compared to controls, we understand the reviewer’s concern. Fluorescence intensity measurements in Fiji provide the mean pixel intensity normalized for the region of interest/area. Therefore, fluorescence intensity values are not influenced by potential size differences between NMJs. We have not measured HRP fluorescence intensity. Our measurements of FM 1-43FX signal intensity are consistent with other publications ^1-3^.

4) Figure 1: On what basis were the 10 genes selected to be analyzed by qPCR? Some explanation would be appreciated. While the results for sgg are convincing, three data points that raise the mean dominate the results for PI3K92E and synd, especially for the Kis13416 mutant. What does a data point mean in this context? Does one data point represent one animal/larva? And why is the number of data points different for each gene?

The gene products were selected because of their roles in ADBE and/or CME as described in the results. The data points represent technical replicates and this information has been added to the figure legend. There are 9-12 data points for each gene product. The number of data points/technical replicates vary based on the number of biological replicates (3-4) and whether individual reactions produced product.

5) Figure S1: Why did BAPTA and EGTA not have an effect on CME or ABDE in control animals? Since they are supposed to inhibit CME and/or ABDE, the finding is surprising.

We were also surprised by these results. While BAPTA showed decreased eEJC amplitudes during both low and high frequency stimulation, these reductions were not statistically significant. We followed published protocols for the duration of exposure and these are referenced in the text.

6) Figure 4 and 5: The presentation of the data (graphs) is very confusing. Here, the untreated and treated genotypes need to be compared to assess how strong the effect of the treatment is in WT and mutants and to judge if the treatments have any effect compared to the respective DMSO control.

We agree that there is more than one way to present these data given that both the compounds and genotypes represent the independent variables for these experiments. The experiments were performed one compound at a time instead of one genotype at a time. Therefore, the graphs shown in Figures 4 and 5 quantify mean relative FM 1-43FX intensities for each compound used. The images shown and data obtained for these experiments were performed on the same days. Comparisons of compounds across genotypes were performed on separate days but, given that the same number of controls were used each day, we created a separate set of graphs to compare the results on a compound by compound basis and these data are shown in Fig S3. These data show that all compounds impaired endocytosis in *w^1118^* controls.

7) Figure 7D: The authors conclude from the data shown that Kis functions in all tissues and neurons to promote proper local locomotion. However, reexpression of Kis in postsynaptic muscle increases larval movement while the reexpression in other tissues had no effect. How does that support the conclusion? Also, why is the control group KisLM27/Kisk13416 missing in this graph? It is very confusing to have to jump to the controls of Figures 8 and 9 first to understand Figure 7D.

We thank the reviewer for pointing out this discrepancy in the text. We have amended Figure 7 to include the *UAS-Kis-L* data (see 8 below) and the *kis^LM27^/kis^k13416^* mutant along with its control, *w^1118^*. We did not include *kis^LM27^/kis^k13416^* as a control for statistical comparisons, however, because the transheterozygous mutant *kis^LM27^/kis^k13416^* is not a control for the reexpression experiments. Reexpression of Kis requires introducing the *UAS-kis-L* transgene in the *kis^LM27^* mutant background and introducing the *Gal4* drivers, which are transgenes, in the *kis^k13416^* background. The chromosomal location of the transgenes can affect phenotypes depending on their respective insertion sites in the genome and there can be “leaky” expression of transgenes ^4,5^. Therefore, given that the presence of transgenes in the genome may produce phenotypes ^4,5^, outcrossed controls (the *kis* stocks including either the *UAS* or the *Gal4* crossed with *w^1118^* controls) represent the closest isogenic controls for *kis* mutants expressing Kis in a specific tissue.

Our data shows that reexpression of Kis in all tissues but not presynaptic motor neurons or postsynaptic muscle increases the distance traveled indicating that *kis* expression in all tissues is important for larval locomotion. We have amended the text to indicate this and thank the reviewer for catching this error.

8) Line 369: UAS-kis-L data should be shown.

These data have been added to Figure 7.

9) Line 451: Please show the unpublished data as supplement

These data are included in another manuscript and, therefore, cannot be shown here. We amended the text to omit the information that referenced “data not shown”.

**Reviewer #2:** This paper extends prior work from the lab showing roles for CHD genes in endocytosis by examining the role of the Drosophila kismet in synaptic vesicle recycling. The authors report differences in both clathrin mediated endocytosis and a backup endocytic pathway, activity dependent bulk endocytosis and then use tissue-specific rescue and knock down experiments to try to tease out the where kismet is required for its functions, with results pointing to a role in post-synaptic muscles. Further ATPase- mutants of kismet rescues both endocytosis and movement dysfunction in the mutant whereas human CHD7 only rescues movement functions. Overall, the study adds to our understanding of this large protein and its novel role in synapses. The discussion is thought provoking and contextualizes the work.

Prior to publication, I have major issues that need to be addressed, most important of which is related to Figures 4 and 5 and the use of drugs and their interpretation:

In figure 4, stars for significance are hard to distinguish from plotted data.

We have moved the histograms for Figures 4 and 5 below the panels showing the raw data to address the comment by the third reviewer. We agreed that the histograms were too small making it difficult to discern the stars for significance and read the text of the graphs.

The quantification in the graphs does not appear to match the images shown, which present a much greater than 2-fold reduction in intensity. More representative average images should be shown.

All images to construct the figures were selected because the image was the closest to the mean relative FM 1-43FX intensity. We have amended the figure by choosing different representative images for some genotypes and conditions.

Furthermore it is unclear how the ratios are assessed here. They state that they are normalizing all of the samples to w1118 but the exposure cases should be normalize to the vehicle control to determine if the pre-treatment has an effect. In that case, it would appear (from the images provided) that kis is affected by Roscovitine but not EGTA or BAPTA, but dap is only affected by BAPTA and for Iqf and sgg it is unclear because the controls are already very weakly green. The interpretation that this shows kis affects just ADBE seems unjustified. I have similar concerns about the images in Figure 5 and the quantification.

We included additional graphs to show the data normalized to the vehicle control for each genotype. These data were requested by Reviewer 1 and are shown in Figure S3. Our interpretation is that Kis affects both ADBE and CME as evidenced by *kis* mutants not exhibiting further reductions in endocytosis when BAPTA and EGTA were used to inhibit ADBE and when Chlorpromazine and Dynasore were used to inhibit CME. These data are now shown in Figures 4, 5 and S3.

More detail should be provided about the molecular nature of each inhibitor and what it does. It is unclear why different mutants would respond differently to inhibitors of the same process.

Although each of these inhibitors were previously used to inhibit ADBE and/or CME, there is no indication in the literature why the inhibitors would differentially affect the same genotypes. We could speculate that the affinity of the inhibitor for its molecular target and the penetrance of the mutant alleles may contribute to these differential affects.

Fig 2. BAPTA and Dynasore are used on the controls, but only the latter on the mutants to show involvement in CME. Both should be tested on the mutants to corroborate the the findings.

We think the reviewer is referring to Fig S2, which shows that none of the compounds used, BAPTA, Dynasore, nor EGTA impair endocytosis in *w^1118^* controls. Endocytosis was induced by high frequency stimulation (20 Hz for one minute) and evoked eEJC amplitudes were recorded. Because none of the compounds impaired endocytosis in controls, they were not used on the mutants.

In figure 2A, the curves for kis and Iqf practically overlap but it is shown that only kis mutants are statistically different than control. This is not at all apparent why this would be the case, especially since we do not see error bars for each time point. I think expanding the Y axis in the region from 50-85 would be helpful to see the differences that are being claimed.

We appreciate this suggestion and made several iterations of this figure prior to submission. Because of the proximity between the curves, adding error bars made it impossible to distinguish one line from another. It was also difficult to expand the Y-axis in the region specified as it distorted the rest of the graph. We revised the Figure so that the 2A panel is larger. This makes it easier to delineate the curves relative to one another.

Again in figure 7, it seems to me that the statistical differences should be tested between the kis heterozygote and homozygote with the gal4 tissue-specific driver not compared with the UAS-kis-L pan driver. It is also unclear why the kis/+; 24B-GAL4/+ has reduced endocytosis in the first place. It suggests that there is a defect in endocytosis caused by the Gal4 drives itself.

We appreciate these suggestions. We did not statistically compare *kis^LM27^/kis^k13416^* mutants with *kis^k13416^* homozygotes with *Gal4* drivers partly because the stocks containing *Gal4* drivers are not homozygous for the Gal4 drivers. In addition, both *UAS-kis-L* and the *Gal4* drivers are transgenes. As described above (Reviewer 1 #7), transgene insertion sites can affect phenotypes depending on their respective insertion sites in the genome. Further, there can be “leaky” expression of transgenes ^4,5^ and these potential influences on phenotypes are not present in *kis^LM27^/kis^k13416^* mutants. Therefore, given that the presence of transgenes in the genome may produce phenotypes ^4,5^, outcrossed controls (the *kis* stocks including either the *UAS* or the *Gal4* crossed with *w^1118^* controls) represent the closest isogenic controls for *kis^LM27^/kis^k13416^* mutants expressing Kis in a specific tissue.

Minor details that should be addressed:

It should be stated that sgg is on the X chromosome necessitating the use of females since non-fly readers do not know that the first chromosome is the X.

This is a good point. The manuscript includes that information in the first section of the methods.

Significance is not marked in Figure 1. Are any significantly different from controls?

Statistical analyses are problematic with n<5 ^6^. All RT-qPCR experiments included 3-4 biological replicates. Each biological replicate includes 30 central nervous systems. Three technical replicates were performed for each biological replicate. Because all three technical replicates used the same pool of RNA, they aren’t considered independent samples. Therefore, we have not previously published statistical analyses of our RT-qPCR data ^2,7^ and this is consistent with recent publications that also show RT-qPCR data but do not use t-tests to analyze those data ^8,9^.

It is stated that “Recovery from HFS was impaired in kis mutants from 40-50 seconds after HFS 240 and at 40 seconds after HFS in lqf mutants,” but the significance starts show differences at 25s and 30s and respectively.

Recovery from high frequency stimulation (HFS) occurs after the HFS stimuli ceased. Thus, the significant differences at 25 and 30 sec occurred during HFS. Once the HFS stimuli ceased, both *kis* and *lqf* mutants exhibited impaired recovery as evidenced by diminished evoked eEJCs at 40-50 and 40 sec, respectively, after HFS. We added a phrase in the text to clarify this.

Line 358, “Similary” should be “Similarly”

Line 426 “for both endocytosis”. Only one item is listed. Remove “both”
Line 437. I think you mean Figs 4,5 not 5,6

Line 480-481: “This circuit may be initiated independent of, but is modulated by, sensory feedback”. needs punctation as marked
We thank the reviewer for finding these typos. We have corrected them.

Are scale bars the same for all images in Figs 4,5, 7? Were these slides imaged with identical settings?

Yes, the scale bars indicate 5 µm. Slides/larvae were imaged by taking the mean of settings used for all controls and applying those means to image all experimental animals on the same day. This is described in the methods section under “Immunocytochemistry and FM Labeling”.

Reviewer #3: In this study, Hendricks and Liebl assess roles for Drosophila Kismet, a chromodomain family (CHD) protein, in regulating presynaptic endocytosis. Alleles of the mammalian homologs of Kis (CHD7 and CHD8) are linked to autism spectrum disorders and CHARGE syndrome, and are thought to regulate a variety of cell functions including cell adhesion and endocytosis, possibly through chromatin remodeling and/or transcriptional regulation. Using reduction-of-function alleles, the authors report that Kis participates in clathrin-mediated and clathrin-independent endocytosis. Surprisingly, its expression in postsynaptic cells appears to be responsible for its role in presynaptic endocytosis, although the mechanisms are currently unclear.

The study itself presents novel and interesting observations for roles of Kis in endocytosis and, even though they do not yet hint at the mechanism of action, this work should be a relevant beginning point for future work examining relationships between CHD proteins, endocytosis and/or regulation of the synaptic vesicle pool, and neuronal function. In places, the authors report counterintuitive findings but do not provide explanations, and as a result many aspects of this manuscript were difficult to follow. The manner in which data were presented in the figures also created challenges.

Specific comments:
1. Many of graphs were very difficult to interpret when printed at page size. Making the graphs larger or increasing font size of axis labels would be helpful.

We appreciated this suggestion and revised Figures 4 and 5. We also revised Figures 2, 3, and 7 to make graphs larger.

2. Throughout the manuscript, the authors distinguish between two forms of endocytosis: clathrin-mediated (CME) and activity-dependent bulk endocytosis (ADBE, which is clathrin-independent). Even in the abstract, the authors state that endocytosis occurs via these two mechanisms, but this is an oversimplification as there are numerous clathrin-independent endocytic pathway. At neurons, ultrafast endocytosis is a clathrin-independent pathway that is distinct from ADBE and which appears to play a prominent role in replenishment of the SV pool. Even if the focus of this study is on CME versus ADBE, the authors should broaden their discussion of endocytic pathways throughout the manuscript to include the likelihood that other pathways are also involved.

Although we acknowledge that other forms of endocytosis occur at synapses, we only alluded to this in the introduction and discussion. We have amended these descriptions in both locations and revised the abstract to mention the other forms of endocytosis.

3. In Figure 1, the authors describe changes in expression for genes linked to CME, ADBE, or both using a kis hypomorph (k13416) and a loss-of-function (heterozygous LM27/k13416). They report 50% reductions of 150% increases in expression for some genes compared to WT, but the significance (statistical and biological, especially since transcriptionally inactive Kis remains functional for endocytosis and locomotion in their later analyses) is not clear. Statistical analyses should be performed to compare the LM27/k13416 to WT controls and to the k13416 hypomorph.

We appreciate this suggestion. See our response to reviewer 2 above.

4. The curves in Figs. 2A and 3A, with accompanying explanation, were confusing. In the text (lines 239-40), the authors state that “recovery from HFS was impaired in kis mutants from 40-50 seconds after HFS and at 40 seconds after HFS in lqf mutants”. Later (lines 253-5), the authors state that kis mutants exhibit a more severe reduction than lqf mutants. In both cases, the wording in the text implies differences between the two mutants, but curves for kis and lqf look essentially identical to one another. At the least, the wording is imprecise and should be modified, but as it stands the data do not support the authors’ conclusions. The curves (as well as those in Figs. 2C, S1 and S2) lack error bars that would add context. Related to this, the fact that Lqf (epsin) is directly involved in CME as an adaptor, and its dephosphorylation triggers ADBE, suggests that it plays roles in both processes. As a result, it is difficult to interpret data using lqf mutants in the context of separating roles in CME versus ADBE.

We thank the reviewer for pointing out this wording. We have revised the text so that there are no implied differences between *kis* and *lqf* mutant responses to HFS and low frequency stimulation. We chose to use *lqf* mutants for the very reason the reviewer describes. We hypothesized that Kis may be important for both CME and ADBE. To begin to test this hypothesis, we needed to compare *kis* mutants to another mutant that also would affect both processes. We chose the *lqf* mutant because of its roles in CME and ADBE as described in the results. Subsequent experiments using chemical inhibitors and additional mutants were designed to assess either CME or ADBE.

5. The authors use Dynasore to inhibit dynamin, with the aim of blocking CME. In Figures S1 and S2, they report that Dynasore inhibited CME but not ADBE. This is surprising because there is a solid body of evidence in the literature demonstrating that dynamin is involved in both endocytic pathways [e.g., Winther et al. (2013) J Cell Sci 126:1021-31 and others]. The reported effect on CME (Fig. S1) is surprisingly small and not consistent across time. Without error bars on these plots it is really difficult to assess whether there is or is not a difference, and the small magnitude of change is not convincing.

Yes, Dynasore inhibited CME and its effect is quite robust. The most pronounced effect was observed from 160-250 sec. During this time, control eEJCs were 75.6-77.7% of the first response. In contrast, Dynasore treatment produced eEJCs that were 57.8-64.1% of the first response. We added this text to the results. We were also surprised that Dynasore along with BAPTA and EGTA did not affect eEJC amplitudes using a stimulation paradigm that induces ADBE. As described earlier, each of these inhibitors were previously used to inhibit ADBE (see text for references). We used the same pretreatment times and conditions as published protocols but did not observe changes in ADBE.

6. The finding that postsynaptic Kis is required for presynaptic endocytosis is interesting, but counterintuitive. The authors speculate extensively about possible explanations, but do not offer any experimental evidence to support any of these explanations. As a result, these results are descriptive. In the final paragraph of the discussion (lines 533-4), the authors state that “our data begins to uncover potential mechanisms by which aberrant chromatin remodeling affect synaptic processes,” but this study is not mechanistic in nature and this statement should be re-phrased.

We rewrote this sentence to, “Our data begins to uncover potential correlates between aberrant chromatin remodeling and synaptic processes.” We do, however, present experimental evidence to explain why postsynaptic Kis may regulate presynaptic endocytosis. Endocytosis occurs adjacent to cell adhesion molecules ^10^, which link the cells of the synapse to downstream signaling pathways and their actin cytoskeletons. Kis restricts the localization of the cell adhesion molecule, FasII ^11^, and this may restrict endocytosis. Further, retrograde signaling impacts many presynaptic processes ^12,13^.

7. The methods do not adequately describe how FM1-43 uptake was quantified. Specifically, did the authors correct for size of the regions they measured?

See response to Reviewer 1 #3.

8. The graphs for Fig. 4 are missing labels to identify the samples with blue data points.

The labels were present but may have been overlooked due to the size of the graphs as this reviewer pointed out. We have increased the size of the graphs in both Figures 4 and 5.

9. Wording of the text accompanying Fig. 7 was extremely confusing, and the experiments appear to lack some controls. This section may be difficult to follow for someone unfamiliar with Drosophila genetics. The data shown in Fig. 7 (images and graphs) needs to include WT and kis controls as a frame of reference for the re-expression experiments; moreover, a Kis-restored UAS should be included to show results for whole-organism re-expression. Statistical analyses should be performed relative to WT or kis mutant controls, in addition to the driver-specific controls. Why do the authors see significance compared to the UAS control but not compared to the driver-specific control in Fig. 7B?

We also anticipated the challenge for individuals in understanding Drosophila genetics. This is why we included “*UAS* outcrossed control”, “neuron outcrossed control”, and so on in the panels of the figure. The purpose of the experiments shown in Figure 7 was to identify the tissue-specific requirements for Kis’ role in endocytosis. Given the amount of data already shown in the figure, we chose to omit the restoration of Kis expression in all tissues of *kis* mutants because those genotypes (one experimental animal and one outcrossed control) do not help identify tissue-specific requirements. *w^1118^* controls and *kis* mutants are not isogenic controls for *kis* mutants expressing Kis in specific tissues as described above in the response to Reviewer 1 #7.

10. On lines 357-8, the authors describe Kis knockdown from a previous study in wing discs, but they should also include confirmation and quantification of knockdown in their experimental setup.

We have added the data for knockdown of Kis in all tissues to the manuscript. We don’t have quantification data, however, for neuron-specific, muscle-specific, or glial-specific knockdown. This is because it is not possible to separate neuronal tissue (including glia) from muscle at the *Drosophila* NMJ because of the structure of the NMJ. The presynaptic motor neurons innervate muscles by forming boutons between muscles and these boutons are buried within muscle tissue.

11. The finding that transcriptionally-inactive Kis restores endocytosis and locomotor behavior (Fig. 9) is surprising and interesting. Immediately after presenting these results, the first paragraph of the discussion states that “our data indicate that Kis promotes both CME and ADBE, possibly by regulating the expression of gene products that mediate these processes” (lines 424-5). It is true that the authors report changes in gene expression in Fig. 1 (although statistical analyses were not presented as noted above), but this statement disagrees with the data from Fig. 9, which suggest that Kis acts independently of its role in regulating gene expression. There is an entire section of the discussion (lines 487-508) devoted to the possible role of Kis in transcriptional regulation of endocytosis, but this may not be relevant given the authors’ findings.

We thank the reviewer for pointing out these sentences in the text. Initially, these sentences may seem contradictory and we have, therefore, revised them. We hypothesize that Kis directly promotes the expression of gene products required for endocytosis while indirectly affecting the expression and/or localization of other gene products required for endocytosis and/or synaptic function. We demonstrated the former by showing that Kis binds both within the promoter and within 200 bp of the transcription start site of *dap160* and *endoB* ^7^. Kis may indirectly influence the expression and/or localization of other endocytic gene products via several mechanisms, some of which are described in the discussion. First, chromatin remodeling enzymes indirectly influence covalent posttranslational modifications of DNA by assembling in complexes with histone modifying enzymes ^14^. Second, Kis may influence the localization of some synaptic proteins because it directly affects the expression of scaffolding proteins and cell adhesion molecules ^11^. Finally, CHD proteins are localized to nucleus, nucleolus, and cytoplasm where they may have context-dependent functions ^15^.

References

1	Guan, Z., Quinones-Frias, M. C., Akbergenova, Y. & Littleton, J. T. Drosophila Synaptotagmin 7 negatively regulates synaptic vesicle release and replenishment in a dosage-dependent manner. *Elife* **9** (2020). <https://doi.org:10.7554/eLife.55443>

2	Hendricks, E. L., Smith, I. R., Prates, B., Barmaleki, F. & Liebl, F. L. W. The CD63 homologs, Tsp42Ee and Tsp42Eg, restrict endocytosis and promote neurotransmission through differential regulation of synaptic vesicle pools. *Front Cell Neurosci* **16**, 957232 (2022). <https://doi.org:10.3389/fncel.2022.957232>

3	Heo, K. *et al.* The Rap activator Gef26 regulates synaptic growth and neuronal survival via inhibition of BMP signaling. *Mol Brain* **10**, 62 (2017). <https://doi.org:10.1186/s13041-017-0342-7>

4	Evangelou, A. *et al.* Unpredictable Effects of the Genetic Background of Transgenic Lines in Physiological Quantitative Traits. *G3 (Bethesda)* **9**, 3877-3890 (2019). <https://doi.org:10.1534/g3.119.400715>

5	Haruyama, N., Cho, A. & Kulkarni, A. B. Overview: engineering transgenic constructs and mice. *Curr Protoc Cell Biol* **Chapter 19**, Unit 19 10 (2009). <https://doi.org:10.1002/0471143030.cb1910s42>

6	de Winter, J. C. F. Using the Student's t-test with extremely small sample sizes. *Practical Assessment, Research and Validation* **18** (2013). <https://doi.org:doi.org/10.7275/e4r6-dj05>

7	Latcheva, N. K. *et al.* The CHD Protein, Kismet, is Important for the Recycling of Synaptic Vesicles during Endocytosis. *Scientific Reports* **9**, 19368 (2019). <https://doi.org:10.1038/s41598-019-55900-6>

8	Borg, R., Herrera, P., Purkiss, A., Cacciottolo, R. & Cauchi, R. J. Reduced levels of ALS gene DCTN1 induce motor defects in Drosophila. *Front Neurosci* **17**, 1164251 (2023). <https://doi.org:10.3389/fnins.2023.1164251>

9	Dulac, A. *et al.* A Novel Neuron-Specific Regulator of the V-ATPase in Drosophila. *eNeuro* **8** (2021). <https://doi.org:10.1523/ENEURO.0193-21.2021>

10	Grossier, J. P., Xouri, G., Goud, B. & Schauer, K. Cell adhesion defines the topology of endocytosis and signaling. *EMBO J* **33**, 35-45 (2014). <https://doi.org:10.1002/embj.201385284>

11	Ghosh, R. *et al.* Kismet positively regulates glutamate receptor localization and synaptic transmission at the Drosophila neuromuscular junction. *PLoS One* **9**, e113494 (2014). <https://doi.org:10.1371/journal.pone.0113494>

12	Marques, G. & Zhang, B. Retrograde signaling that regulates synaptic development and function at the Drosophila neuromuscular junction. *Int Rev Neurobiol* **75**, 267-285 (2006). <https://doi.org:10.1016/S0074-7742(06)75012-7>

13	Suvarna, Y., Maity, N. & Shivamurthy, M. C. Emerging Trends in Retrograde Signaling. *Mol Neurobiol* **53**, 2572-2578 (2016). <https://doi.org:10.1007/s12035-015-9280-5>

14	Jiang, D., Li, T., Guo, C., Tang, T. S. & Liu, H. Small molecule modulators of chromatin remodeling: from neurodevelopment to neurodegeneration. *Cell Biosci* **13**, 10 (2023). <https://doi.org:10.1186/s13578-023-00953-4>

15	Alendar, A. & Berns, A. Sentinels of chromatin: chromodomain helicase DNA-binding proteins in development and disease. *Genes Dev* **35**, 1403-1430 (2021). <https://doi.org:10.1101/gad.348897.121>
