# Supplementary material for: The CHD family chromatin remodeling enzyme, Kismet, promotes both clathrin-mediated and activity-dependent bulk endocytosis
Source: PLoS One. 2024 Mar 21;19(3):e0300255. doi: 10.1371/journal.pone.0300255 (PMC10956772; doi:10.1371/journal.pone.0300255)
Supplement: Attachment — Submitted filename: Response to Reviewers #2.docx [file pone.0300255.s008.docx]

We thank the reviewers for reviewing our manuscript for a second time. We have addressed each of the points below by providing additional explanation, justification based on published literature, and highlight revisions to the manuscript. Reviewer comments are shown in normal text. Our responses are in blue.

Reviewer #2: In general, I feel that the authors minimize a number of the concerns that the reviewers raised initially:
Our intention was not to give the impression that we were minimizing the concerns of the reviewers. Instead, we provided a rationale for the results and representation of data. We often refer to published literature to emphasize that we are not arbitrarily making decisions about our results and representation of data. Instead, we consult the literature to ensure we don’t deviate from recent published standards.

Multiple reviewers bring up the point that dynasore does not affect ADBE. While the authors state that “they are also surprised”, they do nothing to address this concern except state that they followed published protocols. This is not acceptable. They need to resolve why they are getting different results… this aloofness calls into question their experimental findings as a whole.

We did not mean to give the impression that we are lackadaisical but we attributed potential differences to the lack of more literature using Dynasore in flies and/or the challenge of data reproducibility. There are published reports that Dynasore inhibits activity-dependent bulk endocytosis (ADBE) in mammalian in vitro experiments as evidenced by a decrease in dextran import in cultured hippocampal [1] and cerebellar granule neurons [2]. The reports of Dynasore use in flies are largely limited to clathrin-mediated endocytosis [3, 4] but Dynasore was shown to inhibit ADBE in adult fly brains as indicated by inhibited dextran import in surface glia [5]. The challenge of reproducing data has been recognized in the scientific community for over a decade [6]. We strive to mitigate this challenge by ensuring biological replicates represent the variation that is inherent in any phenotype [7].

Rev #1 comment 6. They also do not address the concern here about comparison to the DMSO control.
Reviewer 1’s comment was, “The presentation of the data (graphs) is very confusing. Here, the untreated and treated genotypes need to be compared to assess how strong the effect of the treatment is in WT and mutants and to judge if the treatments have any effect compared to the respective DMSO control.” This comment was made in reference to Figures 4 and 5. Therefore, in response to Reviewer 1’s comment, we added Fig S3, which shows each condition compared to its DMSO control for each genotype. The data corresponding to Figures 4 and 5, then, are shown both on a compound by compound basis (Fig 4, 5) and a genotype by genotype basis (Fig S3).

The response to Rev #2 Fig S2 is not clear. In this case they say there are no effects to ANY of the compounds but Dyasore is used on the mutants. This needs to be clarified.
Fig S2 specifically assesses ADBE, not clathrin mediated endocytosis (CME). Dynasore was only used in experiments to inhibit CME (Fig 3) because we showed in Fig S1 that Dynasore impairs CME induced by 5 Hz stimulation in HL-3 containing 1.0 mM Ca^2+^. This is specified in the results, “We also used Dynasore, an inhibitor of Dyn GTPase activity [8], to block CME.”

Rev 2 comment about molecular nature of compounds. These discussion points need to be added to the text.

We added additional description of each compound in the text of the results.

BoAll reviewers had issues with controls for Fig 7. They must be compare with the Gal4 driver. While the reviewers consider the outcrossed control the most isogenic, their statements about effects of insertion sites of transgenes, further argues that they data must be compared to the Gal4 driver.

We apologize if we’re misinterpreting the reviewer’s comment but each of the *Gal4* driver controls are included in Figure 7. The *Gal4* driver controls are compared with the tissue-specific experimental animals where the *Gal4* driver is used to express a *UAS* transgene. Although some researchers only show *Gal4* or *UAS* outcrossed controls, we show both. The latter is consistent with most other *Drosophila* publications using the *Gal4-UAS* system (for recent examples see [9-12]) and mitigates the concern of transgene-specific effects. We did not compare the outcrossed controls with *w^1118^* or the wild type *Drosophila* strains, *OR* or *wt-B*, because those comparisons do not answer the experimental questions posed. Further, we could not find any examples of statistical comparisons between *Gal4* or *UAS* outcrossed controls with *w^1118^*, *OR*, or *wt-B*, in the published literature where the goal of the experiment was to uncover tissue-specific expression requirements.

In addressing the need for error bars on the qRT-PCR data, the authors now reveal that the results are with the same pool of RNA. This should actually be done as biological and technical replicates.

Our response to this point was, “All RT-qPCR experiments included 3-4 biological replicates. Each biological replicate includes 30 central nervous systems. Three technical replicates were performed for each biological replicate. Because all three technical replicates used the same pool of RNA…” Thus, there were both biological (at least three) and technical replicates (three). Only the technical replicates, not the biological replicates, used the same pool of RNA, which were isolated from the central nervous systems of 30 larvae.

Reviewer #3: In their revised manuscript, the authors have made a number of substantial improvements in response to the reviewers’ initial comments and concerns. However, many of the concerns raised were not addressed, and significant issues remain:

1. In my original point 3, I expressed concern about analysis of gene expression in Figure 1, and other reviewers expressed concerns about this figure as well. Reviewer 1 asked about differences in the number of data points, which the authors explain as differences in the number of biological replicates (n=3-4). It is not clear why there are differences in the number of biological replicates across samples. To account for day-to-day differences in extraction efficiency and other experimental variables, and to allow comparisons between relative changes, it would seem to make more sense that the analyses be paired and have the same number of biological replicates where each replicate was prepared side-by-side.

The 2-^ΔΔC(t)^ method of quantifying and representing RT-qPCR data, which is described in the methods, controls for the daily potentially confounding variables the reviewer mentions. This method first subtracts the cycle threshold (C(t)) value of the target transcript reaction from the C(t) value for GAPDH to obtain ΔC(t) for each transcript. Then, the difference between the control, *w^1118^*, and *kis* mutant ΔC(t)s was calculated. Thus, 2-^ΔΔC(t)^ yields the fold change of target gene expression in *kis* mutants relative to controls, both of which were normalized to the reference transcript, *GAPDH*. 2-^ΔΔC(t)^ are only calculated using RNA samples isolated the same day with RT-qPCR reactions executed simultaneously, each using 100 ng of RNA. We have added the former information and a reference to the first paper that described the 2-^ΔΔC(t)^ calculation to the methods. We thank the reviewer for pointing out this needed clarification to the methods.

Reviewers 2 and 3 requested statistical analysis for the data in Figure 1, which the authors rebutted in saying that statistical analyses are problematic with n<5. A simple solution to addressing this concern is to perform additional replicate experiments so that their n-value is at least 5, which the authors declined to do. Notwithstanding the issue of having different numbers of biological replicates, which in itself is problematic for the reasons described above, the authors should have repeated this experiment or added the necessary additional trials to the existing data, and performed the requested statistical analyses. As it stands, the authors should not be drawing any conclusions about changes in gene expression if those changes are not supported by rigorous analysis.

We did not perform additional biological replicates because the mean number of biological replicates for RT-qPCR experiments is three. However, we recognized that this mean may have changed since we last published RT-qPCR data and, therefore, searched recent literature for neuronal gene expression RT-qPCR data. Although there are examples in the literature of using two biological replicates (with three technical replicates per biological replicate), these are likely due to the scarcity of vertebrate tissue samples (see for example [13]). All other recent publications we examined used three biological replicates. We did, however, find one paper that used four biological replicates with three technical replicates per biological replicate [14]. Therefore, we added an additional biological replicate to our data and have amended Figure 1 to show this. We also found examples of statistical analyses of RNA fold changes [13, 15] and have added this to Figure 1.

2. In my original point 4, I expressed concern about the curves shown in Figs. 2A and 3A. One of the original concerns was that there were no error bars provided (as well as in other curves, e.g., Figs. 3C, 6A, 6B, S1, and S2), and these were not included in the revised manuscript. Reviewer 1 also commented on the lack of error bars for these experiments. Part of the importance of including them is that significance is reported for a very limited number of time points, often with surrounding times that have no reported significance. An alternative interpretation of these results might be that overall recovery is “on the cusp” of being statistically significant, where random noise pushes some time points toward being significant (or pushes some time points toward being not significant). This raises questions about whether there are overall changes in the curves. Regardless, error bars or confidence ranges need to be provided for all curves.

We apologize for not including error bars in our first revision. As we described, inclusion of the error bars makes it challenging to delineate the different points of the conditions/genotypes. We agree with the reviewer that error bars are important and have revised Figures 2, 3, 6, S1, and S2 to include these data.

3. In my original point 5, I again expressed concern about the lack of error bars in figures S1 and S2, where the close proximity of data points made it difficult to assess the significance of relative changes. This point was not addressed.

We revised Figures S1 and S2 to include error bars.

4. In my original point 9, I commented that additional control images and quantification needed to be included, but they were not. The authors stated that they chose to omit the data for restoration of Kis expression in all tissues because of the amount of data already included in the figure; some of these could have been placed in a supplemental figure, especially if the authors did not feel that the data helped identify tissue-specific requirements.

We agree that these data are ideal for a supplemental figure and thank the reviewer for offering this suggestion. We included the *kis* mutant phenotype, restoration of Kis in all tissues of *kis* mutants, and the RT-qPCR data requested in 5 below in a new supplemental figure, S4 Fig.

5. In my original point 10, I requested confirmation of knockdown in the experiments performed. The authors responded that they added the data for knockdown in all tissues (reported in the text as a 56.2% reduction), but this should be listed as “data not shown” because they did not actually show the result. These data should be added to Figure 7.

These data are included in S4 Fig.

1.	Li YY, Zhou JX, Fu XW, Bao Y, Xiao Z. Dephospho-dynamin 1 coupled to activity-dependent bulk endocytosis participates in epileptic seizure in primary hippocampal neurons. Epilepsy Res. 2022;182:106915. Epub 20220330. doi: 10.1016/j.eplepsyres.2022.106915. PubMed PMID: 35390701.

2.	Clayton EL, Anggono V, Smillie KJ, Chau N, Robinson PJ, Cousin MA. The phospho-dependent dynamin-syndapin interaction triggers activity-dependent bulk endocytosis of synaptic vesicles. J Neurosci. 2009;29(24):7706-17. doi: 10.1523/JNEUROSCI.1976-09.2009. PubMed PMID: 19535582; PubMed Central PMCID: PMCPMC2713864.

3.	Gagliardi M, Hernandez A, McGough IJ, Vincent JP. Inhibitors of endocytosis prevent Wnt/Wingless signalling by reducing the level of basal beta-catenin/Armadillo. J Cell Sci. 2014;127(Pt 22):4918-26. Epub 20140918. doi: 10.1242/jcs.155424. PubMed PMID: 25236598; PubMed Central PMCID: PMCPMC4231306.

4.	Nemetschke L, Knust E. Drosophila Crumbs prevents ectopic Notch activation in developing wings by inhibiting ligand-independent endocytosis. Development. 2016;143(23):4543-53. doi: 10.1242/dev.141762. PubMed PMID: 27899511.

5.	Artiushin G, Zhang SL, Tricoire H, Sehgal A. Endocytosis at the Drosophila blood-brain barrier as a function for sleep. Elife. 2018;7. Epub 20181126. doi: 10.7554/eLife.43326. PubMed PMID: 30475209; PubMed Central PMCID: PMCPMC6255390.

6.	Reproducibility and Replicability in Science. Washington (DC)2019.

7.	Voelkl B, Altman NS, Forsman A, Forstmeier W, Gurevitch J, Jaric I, et al. Reproducibility of animal research in light of biological variation. Nat Rev Neurosci. 2020;21(7):384-93. Epub 20200602. doi: 10.1038/s41583-020-0313-3. PubMed PMID: 32488205.

8.	Kirchhausen T, Macia E, Pelish HE. Use of dynasore, the small molecule inhibitor of dynamin, in the regulation of endocytosis. Methods Enzymol. 2008;438:77-93. doi: 10.1016/S0076-6879(07)38006-3. PubMed PMID: 18413242; PubMed Central PMCID: PMCPMC2796620.

9.	Grice SJ, Liu JL. Motor defects in a Drosophila model for spinal muscular atrophy result from SMN depletion during early neurogenesis. PLoS Genet. 2022;18(7):e1010325. Epub 20220725. doi: 10.1371/journal.pgen.1010325. PubMed PMID: 35877682; PubMed Central PMCID: PMCPMC9352204.

10.	Walkowicz L, Krzeptowski W, Krzeptowska E, Warzecha K, Salek J, Gorska-Andrzejak J, et al. Glial expression of DmMANF is required for the regulation of activity, sleep and circadian rhythms in the visual system of Drosophila melanogaster. Eur J Neurosci. 2021;54(5):5785-97. Epub 20210317. doi: 10.1111/ejn.15171. PubMed PMID: 33666288.

11.	Wang X, Davis RL. Early Mitochondrial Fragmentation and Dysfunction in a Drosophila Model for Alzheimer's Disease. Mol Neurobiol. 2021;58(1):143-55. Epub 20200909. doi: 10.1007/s12035-020-02107-w. PubMed PMID: 32909149; PubMed Central PMCID: PMCPMC7704861.

12.	Zhao B, Sun J, Zhang X, Mo H, Niu Y, Li Q, et al. Long-term memory is formed immediately without the need for protein synthesis-dependent consolidation in Drosophila. Nat Commun. 2019;10(1):4550. Epub 20191007. doi: 10.1038/s41467-019-12436-7. PubMed PMID: 31591396; PubMed Central PMCID: PMCPMC6779902.

13.	Greguske EA, Maroto AF, Borrajo M, Palou A, Gut M, Esteve-Codina A, et al. Decreased expression of synaptic genes in the vestibular ganglion of rodents following subchronic ototoxic stress. Neurobiol Dis. 2023;182:106134. Epub 20230424. doi: 10.1016/j.nbd.2023.106134. PubMed PMID: 37100209.

14.	McSweeney D, Gabriel R, Jin K, Pang ZP, Aronow B, Pak C. CASK loss of function differentially regulates neuronal maturation and synaptic function in human induced cortical excitatory neurons. iScience. 2022;25(10):105187. Epub 20220923. doi: 10.1016/j.isci.2022.105187. PubMed PMID: 36262316; PubMed Central PMCID: PMCPMC9574418.

15.	Tan FHP, Azzam G, Najimudin N, Shamsuddin S, Zainuddin A. Behavioural Effects and RNA-seq Analysis of Abeta42-Mediated Toxicity in a Drosophila Alzheimer's Disease Model. Mol Neurobiol. 2023;60(8):4716-30. Epub 20230505. doi: 10.1007/s12035-023-03368-x. PubMed PMID: 37145377.
