# Supplementary material for: The CHD family chromatin remodeling enzyme, Kismet, promotes both clathrin-mediated and activity-dependent bulk endocytosis
Source: PLoS One. 2024 Mar 21;19(3):e0300255. doi: 10.1371/journal.pone.0300255 (PMC10956772; doi:10.1371/journal.pone.0300255)
Supplement: Attachment — Submitted filename: Response to Reviewer #3.docx [file pone.0300255.s009.docx]

Response to Reviewer

We thank the reviewer for taking the time to carefully review our manuscript. We believe we have addressed all the concerns raised by the reviewer as described in the points below.

Reviewer #3: In this revision, the authors have better addressed the concerns raised by reviewers in the two previous rounds of submission. The data are thus improved and more convincing. I have two minor comments (points 1 and 2 below) that should be addressed based on the latest round of revisions, as well as a concern (point 3 below) about one of the major conclusions drawn (that Kis effects are due to regulation of gene expression). For this conclusion to be convincing, the authors would need to do additional supporting experiments; instead, softening the conclusion and incorporating description/discussion of alternative interpretations of their data, especially with the ATPase-dead mutant of Kis, is necessary.

1. Lines 229-30 are imprecise: as written, the authors state that PI3K92E levels are increased in kis mutants, but this is true only for the LM27/kisk13416 mutant, and not the k13416 hypomorph. The k13416 strain doesn’t seem to be used anywhere else in the paper, so the rationale for its inclusion in this figure is unclear. Importantly, do these two genotypes have the same impact on movement and endocytosis? If so, the fact that only one has a difference in PI3K92E transcript level would imply that the observed change is not important, even if it is statistically significant in LM27/k13416.

We have amended the text to clarify which specific mutants show significant changes in expression. We also show the *kis^k13416^* data for two reasons. First, *kis^k13416^* is historically the most common *kis* allele used in publications, possibly because it is homozygous viable. Thus, once published, these data may allow other researchers to draw more direct comparisons with a phenotype of interest. Second, like *kis^LM27^/kis^k13416^* mutants, *kis^k13416^* mutants also exhibit deficits in endocytosis and reduced synaptic levels of the endocytic proteins Dap160 and EndoB. There are differences, however, in the relative localization of Dynamin (Dyn) to the active zone protein, Bruchpilot (Brp) between *kis^k13416^* and *kis^LM27^/kis^k13416^* mutants. At rest, Dyn is localized to active zones but is clustered at periactive zones after synaptic stimulation [1]. This relocalization of Dyn does not occur *kis^k13416^* mutants while *kis^LM27^/kis^k13416^* mutants show a reversal of this relative distribution. Dyn is closer to periactive zones at rest in *kis^LM27^/kis^k13416^* mutants and then is localized near active zones after stimulation [2]. Collectively, our data indicate that the *kis^LM27^/kis^k13416^* and *kis^k13416^* mutants have similar phenotypes but there are subtle differences between mutants in other phenotypes.

2. Line 525: synd should be removed, since the modified data in Figure 1 no longer show changes in its expression.

We removed synd. We thank the reviewer for catching this error.

3. Overall, the fact that ATPase-dead Kis can still correct movement and endocytosis implies that its role in chromatin remodeling (and therefore in regulating transcription) is not required for its regulation of these phenotypes. The authors pointed out in their first rebuttal that Kis localizes to the nucleus, nucleolus, and cytoplasm. Based on these localizations, the conclusion that roles for Kis in endocytosis are due to transcription (e.g., lines 44, 88-90, 522-44) may be true, but cytoplasmic functions cannot be ruled out and indeed may be important given that direct roles of Kis in chromatin remodeling are not required (based on the ATPase-dead mutant results). As written, the conclusion of transcriptional regulation is too strongly stated, especially in the abstract and introduction. It is suitable to speculate on the possibility of gene expression effects in the discussion, but the authors need to make clear that this is one of several possible explanations, and they should expand on alternative interpretations.

Chromatin remodeling enzymes, like Kis, regulate transcription through more than just the ATPase domain. We have added two paragraphs to the discussion to better describe transcriptional regulation by CHD7, CHD8, and Kis. We better emphasized the importance of chromatin remodeling enzymes as part of multiprotein transcriptional complexes and the functional importance of CHD protein domains and amino acid residues outside of the ATPase domains. Our conclusions in the abstract (line 44) and last paragraph (lines 88-90) of the introduction are focused on the role of Kis in endocytosis. In both conclusions, we propose that Kis “may” and “possibly” promotes endocytosis through transcriptional mechanisms. Thus, we were careful not to strongly conclude a definitive transcriptional role for Kis in endocytosis. We only have direct evidence for Kis regulating endocytosis via transcriptional regulation, however, as Kis binds to the promoter and transcription start sites of *endoB* and *dap160* [3]. We are also careful to recognize that many of the effects of Kis may be indirect e.g. deficient endocytosis may occur due to increased cell adhesion molecules and/or loss of Rab11 in *kis* mutants (see paragraphs 4-5 of the discussion).

Although restoration of endocytosis by expressing a Kis lacking the functional ATPase domain in *kis^LM27^/kis^k13416^* mutants could indicate a cytoplasmic role for Kis, we have no direct evidence for this possibility. Further, there isn’t evidence that can be gleaned from the literature on chromatin remodeling enzymes. Our first rebuttal letter specified that CHD proteins, not Kis, have been detected in nucleus, nucleolus, and the cytoplasm. To our knowledge, there are two published reports of CHD proteins outside the nucleus. CHD9 was detected in the nucleus, nucleolus, and the cytoplasm of osteogenic MBA-15 cells *in vitro* [4] and CHD1 is found in the cytoplasm of mitotic cells once the nuclear envelope broke down [5]. We have not detected Kis in the cytoplasm and there is little, if any, in the nucleolus of ventral nerve cord cell bodies or postsynaptic muscles [3]. Although some chromatin modifying enzymes also posttranslationally modify cytoplasmic proteins, these enzymes include family members that are primarily localized to the cytoplasm or shuttle between the cytoplasm and nucleus. In contrast, multiple studies demonstrate that point mutations outside the ATPase domain of the Kis homologs, CHD7 and CHD8, produce neuronal transcriptional changes. These data indicate that the transcriptional activity of CHD proteins is not restricted to the ATPase domain. Further, inhibition of KDM5 demethylase activity in *Chd8* conditional knock outs partly restores oligodendrocyte precursor cell differentiation [6] indicating that CHD8 partly influences oligodendrocyte precursor cell differentiation by its binding to histone methyltransferases. We have carefully reviewed this information and include additional supporting information in the discussion (see lines 524-569).

1.	Winther AM, Vorontsova O, Rees KA, Nareoja T, Sopova E, Jiao W, et al. An Endocytic Scaffolding Protein together with Synapsin Regulates Synaptic Vesicle Clustering in the Drosophila Neuromuscular Junction. J Neurosci. 2015;35(44):14756-70. doi: 10.1523/JNEUROSCI.1675-15.2015. PubMed PMID: 26538647; PubMed Central PMCID: PMCPMC6605226.

2.	Latcheva NK, Delaney TL, Viveiros JM, Smith RA, Bernard KM, Harsin B, et al. The CHD Protein, Kismet, is Important for the Recycling of Synaptic Vesicles during Endocytosis. Scientific Reports. 2019;9(1):19368. doi: 10.1038/s41598-019-55900-6.

3.	Ghosh R, Vegesna S, Safi R, Bao H, Zhang B, Marenda DR, et al. Kismet positively regulates glutamate receptor localization and synaptic transmission at the Drosophila neuromuscular junction. PLoS One. 2014;9(11):e113494. Epub 20141120. doi: 10.1371/journal.pone.0113494. PubMed PMID: 25412171; PubMed Central PMCID: PMCPMC4239079.

4.	Salomon-Kent R, Marom R, John S, Dundr M, Schiltz LR, Gutierrez J, et al. New Face for Chromatin-Related Mesenchymal Modulator: n-CHD9 Localizes to Nucleoli and Interacts With Ribosomal Genes. J Cell Physiol. 2015;230(9):2270-80. doi: 10.1002/jcp.24960. PubMed PMID: 25689118; PubMed Central PMCID: PMCPMC6363339.

5.	Stokes DG, Perry RP. DNA-binding and chromatin localization properties of CHD1. Mol Cell Biol. 1995;15(5):2745-53. doi: 10.1128/MCB.15.5.2745. PubMed PMID: 7739555; PubMed Central PMCID: PMCPMC230505.

6.	An Y, Zhang L, Liu W, Jiang Y, Chen X, Lan X, et al. De novo variants in the Helicase-C domain of CHD8 are associated with severe phenotypes including autism, language disability and overgrowth. Hum Genet. 2020;139(4):499-512. Epub 20200124. doi: 10.1007/s00439-020-02115-9. PubMed PMID: 31980904.
